# Community health centers and primary care access and quality for chronically-ill patients – a case-comparison study of urban Guangdong Province, China

**DOI:** 10.1186/s12939-015-0222-7

**Published:** 2015-12-14

**Authors:** Leiyu Shi, De-Chih Lee, Hailun Liang, Luwen Zhang, Marty Makinen, Nathan Blanchet, Ruth Kidane, Magnus Lindelow, Hong Wang, Shaolong Wu

**Affiliations:** 1 Johns Hopkins Bloomberg School of Public Health, 624 N. Broadway, Baltimore, MD 21205 USA; 2 Department of Information Management, Da-Yeh University, Changhua, 51591 Taiwan (ROC); 3 Johns Hopkins Primary Care Policy Center, Baltimore, 624 N. Broadway, Baltimore, MD 21205 USA; 4 School of Public Health of Sun Yat-sen University, 74, Zhongshan Road II, Guangzhou, 510275 China; 5 Results for Development Institute, 1100 15th Street, NW, Washington, DC 20005 USA; 6 The World Bank, 1225 Connecticut Avenue NW, Washington, DC 20433 USA; 7 Bill & Melinda Gates Foundation, 500 Fifth Avenue North, Seattle, WA 98109 USA

**Keywords:** Primary care, Chronic disease, Community health centers, Integrated care, Quality of care

## Abstract

**Objective:**

Reform of the health care system in urban areas of China has prompted concerns about the utilization of Community Health Centers (CHC). This study examined which of the dominant primary care delivery models, i.e., the public CHC model, the ‘gate-keeper’ CHC model, or the hospital-owned CHC models, was most effective in enhancing access to and quality of care for patients with chronic illness.

**Methods:**

The case-comparison design was used to study nine health care organizations in Guangzhou, Dongguan, and Shenzhen cities within Guangdong province, China. 560 patients aged 50 or over with hypertension or diabetes who visited either CHCs or hospitals in these three cities were surveyed by using face-to-face interviews. Bivariate analyses were performed to compare quality and value of care indicators among subjects from the three cities. Multivariate analyses were used to assess the association between type of primary care delivery and quality as well as value of chronic care after controlling for patients’ demographic and health status characteristics.

**Results:**

Patients from all three cities chose their current health care providers primarily out of concern for quality of care (both provider expertise and adequate medical equipment), patient-centered care, and insurance plan requirement. Compared with patients from Guangzhou, those from Dongguan performed significantly better on most quality and value of care indicators. Most of these indicators remained significantly better even after controlling for patients' demographic and health status characteristics. The Shenzhen model (hospital-owned and -managed CHC) was generally effective in enhancing accessibility and continuity. However, coordination suffered due to seemingly duplicating primary care outpatients at the hospital setting. Significant associations between types of health care facilities and quality of care were also observed such that patients from CHCs were more likely to be satisfied with traveling time and follow-up care by their providers.

**Conclusion:**

The study suggested that the Dongguan model (based on insurance mandate and using family practice physicians as ‘gate-keepers’) seemed to work best in terms of improving access and quality for patients with chronic conditions. The study suggested adequately funded and well-organized primary care system can play a gatekeeping role and has the potential to provide a reasonable level of care to patients.

## Introduction

Primary care refers to first-contact, continuous, comprehensive, and coordinated care provided to individuals regardless of gender, disease, or organ system affected [1]. Over the past two decades, preponderance of research around the world has demonstrated that effective primary care can not only improve population health, but also has the potential to reduce health disparities [2–12]. Countries or regions within a country with strong primary care system see improved health outcomes for their populations, particular reflected in reduced morbid conditions, higher quality of life, and better health care system satisfaction in elderly individuals [7, 13–17]. In 2008, the World Health Organization (WHO) urged that primary care be used as an approach to provide effective, fair, and efficient care and that primary care systems be strengthened in all countries [18]. Studies in the United States have credited the community health center model with providing accessible, cost-effective, and high quality primary care and reducing health disparities [19–46]. These studies suggest that primary care matters to population health and that the health center model might be an effective approach to advance primary care.

China has a three-tiered health care delivery system, with community health centers (CHCs) at the bottom, secondary hospitals in the middle, and tertiary hospitals at the top [47, 48]. Despite this classification, patients can access primary care in all facilities across the three levels, having the freedom to choose a doctor or health care facility without the constraints of health insurance policy [49]. Most people prefer higher-level hospitals due to better medical technology and perceived technical quality of the provider, although they will not see the same doctor each time and expenditure at the hospital setting is much higher than that at the community. However, higher-level hospitals require registering in a long queue, which has a significant time constraint. This also means that doctors have less time to treat and interact with their patients. This may result in unsatisfactory experience of care, as patients often complain that their doctors hustle them through appointments.

In an attempt to address the access and cost problems associated with seeking hospital care for all health problems, the Chinese government has undertaken a series of reforms aimed at strengthening community-based primary care delivery and the referral system [50]. The central and local governments have been investing heavily in primary care service institutions to provide full funding for their staff and the supply of zero-profit drugs [51, 52]. Numerous models have occurred around the country to enhance community-based primary care. As socioeconomic conditions and health care development vary in different urban regions, the primary care models implemented also differ accordingly. The most popular model is that of an independent CHC fully funded by the government and acting as a first-contact option for patients seeking primary care. The rational is that by providing easy access and affordable care, CHC would attract patients from hospitals. However, under this public CHC model, patients still have the option of choosing hospitals for their primary care needs. Most CHCs in Guangzhou and Beijing are under this model. The Beijing government established Beijing Management Center for Community Health Services (CCHS) in 2006, which served as an executive agency and played the role of drafting and implementing standards and norms for public CHCs around the city. Another more restrictive model uses insurance payment arrangement to require patients to use CHC as their first-contact ‘gate-keeper.’ Dongguan is the first pilot city implementing ‘gate-keeper’ model since 2008. A third emerging model focuses on the integration of hospital and CHC where CHC serves as extension of the hospital. In this model, CHC is staffed and managed by the hospital. The rationale is that by making the CHC part of the hospital, the technical expertise of the doctors can be assured and a seamless referral (between CHC and hospital) can be facilitated. Implementation of the hospital-owned CHC model began in Shenzhen and was expanded to Chongqing as well as other cities around the nation. Despite these and other emerging models of primary care delivery, no systematic research has been conducted to assess their effectiveness.

The purpose of this study was to fill this gap in the literature by evaluating which of the dominant primary care delivery models are most effective in enhancing access and quality for patients with chronic illness, using the province of Guangdong as the study site. Guangdong is a coastal province located in Southern China. The population of permanent residents in Guangdong is more than 100 million, making it the most populous province in China. With 30 % of its total population being migrants, it accounts for the largest number of internal migrant population in China [53]. Variable economic and geographic development makes Guangdong a good case study for China. Guangzhou is larger in land area and population (3834 square kilometers and 12,927 thousand) compared with Shenzhen (1997 square kilometers and 10,629 thousand) and Dongguan (2465 square kilometers and 8317 thousand). However, GDP per capita was higher in Shenzhen (RMB 136,948) than in Guangzhou (RMB 119,695) or Dongguan (RMB 66,109). In terms of health status, while the three cities had comparable infant mortality (2.2-3.5 per 1000 live births), Shenzhen had lower mortality rate than Dongguan and Guangzhou (1.07 vs. 4.60 and 5.44 %). In terms of health care resources, Shenzhen had more CHCs than Dongguan and Guangzhou (609 vs. 389 and 316). There were more hospital beds and higher bed occupancy rate in Guangzhou (5.67 beds per 1000 population and 91.2 %) compared to Shenzhen (2.76 beds per 1000 population and 84.4 %) and Dongguan (3.09 beds per 1000 population and 88.2).^1^

With a rapid pace in economic development, Guangdong province plays a leading role in trend-setting implementation of health policy initiatives with developed primary care infrastructure [54]. Variations in socioeconomic conditions and differences of local government in Guangdong have led to the emergence of three dominant models of primary care delivery as described earlier: the public CHC model, the ‘gate-keeper’ CHC model, and the hospital-owned CHC model. Thus, Guangdong affords an idea study site to assess the impact of these CHC models. To the extent that patient health seeking behavior is altered and access and quality improved as a result of these models, the Guangdong experience could serve as role model for other urban Chinese provinces as well as other countries striving to improve their primary care delivery. Results of the study could provide implications for policymakers in terms of improving primary care performance in China, and help guide patients in their health care seeking behaviors.

## Methods

The case-comparison method was used to carry out this evaluative study. Specifically, Guangzhou, Dongguan, and Shenzhen cities within Guangdong province were selected due to the implementation of the three CHC models. As is the case with most urban China, Guangzhou has adopted the public CHC model in which government owns and operates the CHC and residents nearby are encouraged (although not required) to use CHC for primary care for convenience and at reduced price. Dongguan has adopted the ‘gate-keeper’ CHC model in which insurance mandates that patients go through CHC (by signing up with a family physician) as their entrance into the health care system and that CHC would coordinate with referrals if necessary. Shenzhen has adopted the hospital-owned CHC model whereby CHC serves as extension of the hospital.

Within each city, we selected two settings as sites for data collection. These included district or secondary hospitals and CHCs. These sites were selected since they were the target health care facilities for the referral system. The rationale is that by including these facilities from the three cities, we will be able to assess which primary care model performs best in terms enhancing access and quality. The selection of study sites was based on purposive sampling, with input from our local research partner, faculty from the School of Public Health at the Sun Yat-sen University. Specifically, one hospital and two CHCs were selected from each city.

### Study subjects

The study subjects were individuals age 50 or over with hypertension or diabetes who visited either CHCs or hospitals in these three cities in March 2015. These two conditions were selected because they are the most common chronic conditions affecting the elderly in China and are most amenable to improved primary care and referral system, and findings from previous research indicated that major chronic conditions were common, so prevention and early intervention targeting adults aged 50 years and older should be prioritized [55].

### Data

Data for this study came from face-to-face interviews with patients, selected in a systematic manner (i.e., every 5th patient that met the selection criteria until the total quota was reached for that site). The sample size was calculated based on findings from a previous paper [56], and adjusted for site specific variations and refusal rate. Based on sample size calculation for survey respondents with 95 % confidence interval, 80 % power, and three locations, a minimum sample size of 80 patients were required for each type of facility (i.e., CHC and hospital), or a total of 480 patients for three cities (i.e., 80 from CHC and 80 from hospital per city). The actual sample size was 560, 80 more patients than minimally required (180 from Shenzhen, 200 from Dongguan and 180 from Guangzhou). Eight graduate students from the local Sun Yat-sen University conducted the face-to-face interview, with on-site supervision from their faculty advisor and the project investigative team from Johns Hopkins University Primary Care Policy Center. Before data collection, we held a one-day training session to set forth guidelines and procedures for the students conducting the interview. In addition, all students were monitored on a pilot test where patients from a non-study site were interviewed to check out the wording of the questions as well as the conduct of the interviews. Upon completion of the interview, each study subject was given a gift of daily necessity (e.g., toothpaste, soap, mug) valued at under $5. The Human Subjects Research Committee of Sun Yat-sen University reviewed and approved the protocol of the study and patient survey data collection in compliance with the Declaration of Helsinki–Ethical Principles for Medical Research Involving Human Subjects.

### Measures

The well-known and widely-used Behavioral Model of Health Services Utilization served as the framework for the study and provided guidance in the selection of measures to carry out the study [57]. According to this framework, health care use is influenced by both individual and system factors. Individual factors consist of predisposing, enabling, and need. Predisposing factors are exogenous factors that influence one’s inclination to use health care services, such as age, gender, occupation, ethnicity, education, and other demographic, social structure, and health belief factors. Enabling factors denote the availability of health care services and the ability of an individual to access services, such as health insurance, income, ability to travel, and distance to the nearest health care institutions. Need factors take health status into account by measuring existing disease, symptoms, general health status, disabilities, and other chronic health conditions. System factors include such characteristics of health care delivery as organizing, financing, and availability, and are reflective of the new models associated with the new healthcare delivery model. Based on the above-mentioned components of the conceptual framework, we extracted independent and covariate measures for this study. We coded gender, marital status, residence status, occupation, education, type of health insurance, health status and chronic condition status as categorical variables, and age and per capita income as continuous variables. These measures as well as the coding method are listed in Table 1.

**Table 1:** Patient characteristics: Shenzhen, Dongguan and Guangzhou

	Shenzhen				Dongguan				Guangzhou			
	Total N	CHC 1-Xiasha N (%)	CHC 2-Zhulin N (%)	Hospital-Futian N (%)	Total N	CHC 1-Shilong N (%)	CHC 2-Liaobu N (%)	Hospital-Liaobu N (%)	Total N	CHC 1-Fengyuan N (%)	CHC 2-Longjin N (%)	Hospital-Liwan N (%)
Sample Size	180	50	50	80	200	50	50	100	180	50	50	80
Gender**												
Male	100 (55.56)	30 (60.00)	23 (46.00)	47 (58.75)	98 (49.00)	19 (38.00)	21 (42.00)	58 (58.00)	68 (37.78)	22 (44.00)	14 (28.00)	32 (40.00)
Female	80 (44.44)	20 (40.00)	27 (54.00)	33 (41.25)	102 (51.00)	31 (62.00)	29 (58.00)	42 (42.00)	112 (62.22)	28 (56.00)	36 (72.00)	48 (60.00)
Age (Mean)**	62.36 (8.86)	58.64 (6.94)	61.90 (6.69)	64.96 (10.22)	65.21 (12.84)	65.32 (10.09)	65.85 (9.78)	64.85 (15.16)	65.36 (9.74)	69.82 (10.25)	64.08 (9.15)	63.36 (8.95)
Marital Status*												
Married	168 (93.33)	49 (98.00)	49 (98.00)	70 (87.50)	182 (91.00)	47 (94.00)	44 (88.00)	91 (91.00)	151 (83.89)	39 (78.00)	44 (88.00)	68 (85.00)
Not married	12 (6.67)	1 (2.00)	1 (2.00)	10 (12.50)	18 (9.00)	3 (6.00)	6 (12.00)	9 (9.00)	29 (16.11)	11 (22.00)	6 (12.00)	12 (15.00)
Residence Status***												
Registered Resident	97 (53.89)	20 (40.00)	33 (66.00)	44 (55.00)	157 (78.50)	49 (98.00)	43 (86.00)	65 (65.00)	136 (75.56)	42 (84.00)	44 (88.00)	50 (62.50)
Non-registered Resident/Migrant	83 (46.11)	30 (60.00)	17 (34.00)	34 (45.00)	43 (21.50)	1 (2.00)	7 (14.00)	35 (35.00)	44 (24.44)	8 (16.00)	6 (12.00)	30 (37.50)
Current Occupation***												
Enterprise	31 (17.22)	17 (34.00)	3 (6.00)	11 (13.75)	13 (6.50)	1 (2.00)	3 (6.00)	9 (9.00)	7 (3.89)	1 (2.00)	1 (2.00)	5 (6.25)
Farmer	9 (5.00)	4 (8.00)	1 (2.00)	4 (5.00)	68 (34.00)	9 (18.00)	12 (24.00)	47 (47.00)	5 (2.78)	1 (2.00)	0	4 (5.00)
Retired	115 (63.89)	21 (42.00)	42 (84.00)	52 (65.00)	86 (43.00)	33 (66.00)	27 (54.00)	26 (26.00)	150 (83.33)	41 (82.00)	43 (86.00)	66 (82.50)
Other	25 (13.89)	8 (16.00)	4 (8.00)	13 (16.25)	33 (16.50)	7 (14.00)	8 (16.00)	18 (18.00)	18 (10.00)	7 (14.00)	6 (12.00)	5 (6.25)
Highest Education***												
Primary school or below	41 (22.78)	11 (22.00)	11 (22.00)	19 (23.75)	93 (46.50)	27 (54.00)	32 (64.00)	34 (34.00)	45 (25.00)	18 (36.00)	13 (26.00)	14 (17.50)
Middle school	39 (21.67)	6 (12.00)	17 (34.00)	16 (20.00)	64 (32.00)	18 (36.00)	14 (28.00)	32 (32.00)	53 (29.44)	19 (38.00)	16 (32.00)	18 (22.50)
High school	47 (26.11)	20 (40.00)	11 (22.00)	16 (20.00)	30 (15.00)	2 (4.00)	3 (6.00)	25 (25.00)	52 (28.99)	8 (16.00)	16 (32.00)	28 (35.00)
Above high school	53 (29.44)	13 (26.00)	11 (22.00)	29 (36.25)	13 (6.50)	3 (6.00)	1 (2.00)	9 (9.00)	30 (16.67)	5 (10.00)	5 (10.00)	20 (25.00)
Per Capita Income RMB (Mean)***	36639.61 (28780.05)	34179.52 (34143.38)	26984.29 (17961.06)	44405.91 (28832.02)	16791.11 (14935.2)	13940.15 (10496.2)	16027.02 (14949.28)	18436.39 (16439.7)	26119 (19619.62)	32195.57 (27726.08)	21312.93 (16445.85)	25340.83 (13898.95)
Type of Health Insurance***												
Urban Social Insurance for Workers	77 (42.78)	13 (26.00)	18 (36.00)	46 (57.50)	24 (12.00)	9 (18.00)	3 (6.00)	12 (12.00)	86 (47.78)	20 (40.00)	13 (26.00)	53 (66.25)
Urban Social Insurance for Residents	48 (26.67)	9 (18.00)	23 (46.00)	16 (20.00)	91 (45.50)	25 (50.00)	25 (50.00)	41 (41.00)	72 (40.00)	27 (54.00)	32 (64.00)	13 (16.25)
Uninsured/Self-pay	14 (7.78)	7 (14.00)	3 (6.00)	4 (5.00)	12 (6.00)	0	2 (4.00)	10 (10.00)	7 (3.89)	1 (2.00)	1 (2.00)	5 (6.25)
Other	41 (22.78)	21 (42.00)	6 (12.00)	14 (17.50)	73 (36.50)	16 (32.00)	20 (40.00)	37 (37.00)	15 (8.33)	2 (4.00)	4 (8.00)	9 (11.25)
^a^Current Health Status***												
Excellent/Very good/Good	82 (45.56)	32 (64.00)	16 (32.00)	34 (42.50)	110 (55.00)	42 (84.00)	23 (46.00)	45 (45.00)	53 (29.44)	17 (34.00)	14 (28.00)	22 (27.50)
Fair/Poor	98 (54.44)	18 (36.00)	34 (64.00)	46 (57.50)	90 (45.00)	8 (24.00)	27 (54.00)	55 (55.00)	127 (70.56)	33 (64.00)	36 (72.00)	58 (72.50)
^b^Chronic Conditions												
Hypertension or high blood pressure**	137 (76.11)	46 (92.00)	45 (90.00)	46 (57.50)	154 (77.00)	41 (82.00)	41 (82.00)	72 (72.00)	114 (63.33)	40 (80.00)	41 (82.00)	33 (41.25)
Diabetes***	62 (34.44)	9 (18.00)	16 (32.00)	37 (46.25)	85 (42.50)	20 (40.00)	18 (36.00)	47 (47.00)	104 (57.78)	16 (32.00)	11 (22.00)	77 (96.25)
Heart disease*	43 (23.89)	2 (4.00)	11 (22.00)	30 (37.50)	32 (16.00)	8 (16.00)	5 (10.00)	19 (19.00)	49 (27.22)	13 (26.00)	19 (38.00)	17 (21.25)
Joint pain or arthritis***	33 (18.33)	4 (8.00)	22 (44.00)	7 (8.75)	25 (12.50)	7 (14.00)	10 (20.00)	8 (8.00)	56 (31.11)	18 (36.00)	16 (32.00)	22 (27.50)
Other	17 (9.44)	5 (10.00)	5 (10.00)	7 (8.75)	11 (5.50)	0	8 (16.00)	3 (3.00)	9 (5.00)	3 (6.00)	4 (8.00)	2 (2.50)
Lung problems	11 (6.11)	1 (2.00)	6 (12.00)	4 (5.00)	15 (7.50)	1 (2.00)	0	14 (14.00)	15 (8.33)	4 (8.00)	7 (14.00)	4 (5.00)
Stroke*	4 (2.22)	0	1 (2.00)	3 (3.75)	14 (7.00)	5 (10.00)	2 (4.00)	7 (7.00)	4 (2.22)	3 (6.00)	1 (2.00)	0
Cancer	1 (0.56)	0	0	1 (1.25)	3 (1.50)	0	1 (2.00)	2 (2.00)	5 (2.78)	1 (2.00)	1 (2.00)	3 (3.75)
Mental health problems	0	0	0	0	1 (0.50)	0	0	1 (1.00)	2 (1.11)	0	2 (4.00)	0

^a^Participants were asked to answer with a 5-category Likert response scale (excellent, very good, good, fair or poor)

^b^This variable is a patient’s self-reported measure with a Yes/No response, worded in the questionnaire as follows: Have you ever been told by a doctor or healthcare professional that you have any chronic conditions as listed below

*p < 0.05 **p < 0.01, ***p < 0.001 based on ANOVA continuous measures and Chi square test for categorical measures for the Total among three cities

In addition, we conceptualize four dimensions of quality of primary care services and three aspects of values as represented in Starfield’s model of primary care [13]. The four quality dimensions are: accessibility, continuity, coordination, and comprehensiveness. The three aspects of value are satisfaction, cost, and health improvement. We included three dependent measures from each of the four quality dimensions, and two dependent measures from each of the three aspects of values. The dependent variables were coded as continuous or dichotomous. The continuous measures included: satisfaction with traveling time, satisfaction with accessing out-of-office hours by phone or text message, total score of satisfaction with current care provider, and overall satisfaction with the care experience. These measures were coded as continuous because of the way these questions were asked and relatively equal distributions across the response categories. The other outcome measures were coded as dichotomous due to a clear concentration on few response categories. These outcome measures and coding method are listed in Table 2.

**Table 2:** Quality & value of care: Shenzhen, Dongguan and Guangzhou

	Shenzhen				Dongguan				Guangzhou			
	Total N (%) or Mean (SE)	CHC 1-Xiasha N (%) or Mean (SE)	CHC 2-Zhulin N (%) or Mean (SE)	Hospital-Futian N (%) or Mean (SE)	Total N (%) or Mean (SE)	CHC 1-ShilongN (%) or Mean (SE)	CHC 2-Liaobu N (%) or Mean (SE)	Hospital-Liaobu N (%) or Mean (SE)	Total N (%) or Mean (SE)	CHC 1-Fengyuan N (%) or Mean (SE)	CHC 2-Longjin N (%) or Mean (SE)	Hospital-Liwan N (%) or Mean (SE)
Sample Size	180	50	50	80	200	50	50	100	180	50	50	80
Quality of Care												
Access												
Get medical care in the evenings, on weekends, or holidays*												
Very easy/Somewhat easy	39 (21.67)	11 (22.00)	14 (28.00)	14 (17.50)	44 (22.00)	5 (10.00)	11 (22.00)	28 (28.00)	23 (12.78)	2 (4.00)	6 (12.00)	15 (18.75)
Somewhat difficult/Very difficult/Not sure	141 (78.33)	39 (78.00)	36 (72.00)	66 (82.50)	156 (78.00)	45 (90.00)	39 (78.00)	72 (72.00)	157 (87.22)	48.00 (96.00)	44 (88.00)	65 (81.25)
^a^Satisfaction to Current Care-Provider’s Convenience (traveling time)	4.49 (0.05)	4.50 (0.07)	4.84 (0.07)	4.26 (0.08)	4.51 (0.06)	4.52 (0.10)	4.74 (0.11)	4.39 (0.08)	4.41 (0.07)	4.41 (0.13)	4.58 (0.12)	4.30 (0.10)
^b^Satisfaction to Current Care-Provider’s Accessibility (access out-of-office hours by phone or text message)***	4.27 (0.05)	4.30 (0.07)	4.28 (0.16)	4.24 (0.06)	4.31 (0.06)	4.40 (0.10)	3.88 (0.14)	4.48 (0.06)	3.79 (0.08)	3.36 (0.17)	4.02 (0.14)	3.93 (0.11)
Continuity												
Healthcare professional reviewed with you all the medications***												
Yes	81 (45.00)	29 (58.00)	24 (48.00)	28 (35.00)	124 (62.00)	25 (50.00)	31 (62.00)	68 (68.00)	77 (42.78)	22 (44.00)	12 (24.00)	43 (53.75)
No/Not sure/Decline to answer	99 (55.00)	21 (42.00)	26 (52.00)	52 (65.00)	76 (38.00)	25 (50.00)	19 (38.00)	32 (32.00)	103 (57.22)	28 (56.00)	38 (76.00)	37 (46.25)
Health professionals always encourage you to ask questions***												
Always/ Often	99 (55.00)	33 (66.00)	24 (48.00)	42 (52.50)	127 (63.50)	33 (66.00)	30 (60.00)	64 (64.00)	59 (32.78)	10 (20.00)	17 (34.00)	32 (40.00)
Sometimes/Rarely or never/NA/Not sure/Decline to answer	81 (45.00)	17 (34.00)	26 (52.00)	38 (47.50)	73 (36.50)	17 (34.00)	20 (40.00)	36 (36.00)	121 (67.22)	40 (80.00)	33 (66.00)	48 (60.00)
Healthcare professional contacts you to see how things are going***												
Yes	120 (66.67)	42 (84.00)	27 (54.00)	51 (63.75)	146 (73.00)	39 (78.00)	38 (76.00)	69 (69.00)	66 (36.67)	9 (18.00)	11 (22.00)	46 (57.50)
No/Not sure/Decline to answer	60 (33.33)	8 (16.00)	23 (46.00)	29 (36.25)	54 (27.00)	11 (22.00)	12 (24.00)	31 (31.00)	114 (63.33)	41 (82.00)	39 (78.00)	34 (42.50)
Coordination												
Review the prescription and help patients take their medications correctly (Coordinate medication)***												
Yes	144 (80.00)	44 (88.00)	31 (62.00)	69 (86.25)	191 (95.50)	49 (98.00)	47 (94.00)	95 (95.00)	144 (80.00)	36 (72.00)	40 (80.00)	68 (85.00)
No/Not Sure	36 (20.00)	6 (12.00)	19 (38.00)	11 (13.75)	9 (4.50)	1 (2.00)	3 (6.00)	5 (5.00)	36 (20.00)	14 (28.00)	10 (20.00)	12 (15.00)
Make necessary referrals***												
Yes	51 (28.33)	19 (38.00)	13 (26.00)	19 (23.75)	96 (48.00)	15 (30.00)	24 (48.00)	57 (57.00)	24 (13.33)	6 (12.00)	7 (14.00)	11 (13.75)
No/Not Sure	120 (71.67)	31 (62.00)	37 (74.00)	61 (76.25)	104 (52.00)	35 (70.00)	26 (52.00)	43 (43.00)	156 (86.67)	44 (88.00)	43 (86.00)	69 (86.25)
^c^Experienced coordination problems**												
No	136 (75.56)	36 (72.00)	39 (78.00)	61 (75.25)	184 (92.00)	44 (88.00)	44 (88.00)	96 (96.00)	147 (81.67)	47 (94.00)	34 (68.00)	66 (82.50)
Yes/Not sure/Decline to answer	44 (24.44)	14 (28.00)	11 (22.00)	19 (23.75)	16 (8.00)	6 (12.00)	6 (12.00)	4 (4.00)	33 (18.33)	3 (6.00)	16 (32.00)	14 (17.50)
Comprehensiveness												
Received Secondary Prevention Services**												
Yes	52 (28.89)	17 (34.00)	23 (46.00)	12 (15.00)	88 (44.00)	27 (54.00)	28 (56.00)	33 (33.00)	56 (31.11)	12 (24.00)	10 (20.00)	34 (42.50)
No/Not Sure	128 (71.11)	33 (66.00)	27 (54.00)	68 (85.00)	112 (56.00)	23 (46.00)	22 (44.00)	67 (67.00)	124 (58.89)	38 (76.00)	40 (80.00)	46 (57.50)
Health professionals talked with you about things that can cause stress***												
Yes	71 (39.44)	30 (60.00)	13 (26.00)	28 (35.00)	94 (47.00)	12 (24.00)	30 (60.00)	52 (52.00)	37 (20.56)	8 (16.00)	10 (20.00)	19 (23.75)
No/Have not seen a doctor in past 2 years/Not sure/Decline to answer	109 (60.56)	20 (40.00)	37 (74.00)	52 (65.00)	106 (53.00)	38 (76.00)	20 (40.00)	48 (48.00)	143 (79.44)	42 (84.00)	40 (80.00)	61 (76.25)
Health professionals talked with you about healthy diet or exercise**												
Yes	164 (91.11)	49 (98.00)	45 (90.00)	70 (87.50)	190 (95.00)	48 (96.00)	46 (92.00)	96 (96.00)	148 (82.22)	36 (72.00)	36 (72.00)	76 (95.00)
No/Have not seen a doctor in past 2 years/Not sure/Decline to answer	16 (8.89)	1 (2.00)	5 (10.00)	10 (12.50)	10 (5.00)	2 (4.00)	4 (8.00)	4 (4.00)	32 (17.78)	14 (28.00)	14 (28.00)	4 (5.00)
Value of Care												
Satisfaction												
^d^Total Score of Satisfaction of Current Care Provider***	60.46 (0.46)	60.6 (0.78)	61.51 (1.12)	59.72 (0.57)	61.40 (0.54)	63.46 (0.83)	58.26 (1.39)	61.93 (0.66)	57.42 (0.59)	56.04 (1.16)	57.74 (1.06)	58.08 (0.88)
^e^Overall satisfaction with the care experience*	4.23 (0.06)	4.26 (0.06)	4.05 (0.20)	4.33 (0.06)	4.39 (0.05)	4.44 (0.11)	4.20 (0.12)	4.46 (0.05)	4.16 (0.05)	4.00 (0.09)	4.14 (0.09)	4.28 (0.07)
Cost Concern												
Satisfaction to Out-of-pocket Cost for Chronic Care***												
Sample size	106	24	24	58	168	38	48	82	129	37	33	59
Payment very easily/easily afforded and affordable	32 (30.19)	10 (41.67)	6 (25.00)	16 (27.59)	83 (49.40)	22 (57.89)	32 (66.67)	29 (35.37)	35 (27.13)	17 (45.95)	11 (33.33)	7 (11.86)
Payment too high/way too high	74 (69.81)	14 (58.33)	18 (75.00)	42 (72.41)	85 (50.60)	16 (42.11)	16 (33.33)	53 (64.63)	94 (72.87)	20 (54.05)	22 (66.67)	52 (88.14)
Not receive the help you needed because of the cost												
No	163 (90.56)	47 (94.00)	41 (82.00)	75 (93.75)	170 (85.00)	37 (74.00)	43 (86.00)	90 (90.00)	164 (91.11)	46 (92.00)	47 (94.00)	71 (88.75)
Yes/Not sure	17 (9.44)	3 (6.00)	9 (18.00)	5 (6.25)	30 (15.00)	13 (26.00)	7 (14.00)	10 (10.00)	16 (8.89)	4 (8.00)	3 (6.00)	9 (11.25)
Health Improvement												
Chronic Condition Relative to When it was First Diagnosed**												
Significantly/Somewhat improved	126 (70.00)	48 (96.00)	29 (58.00)	49 (61.25)	168 (84.00)	43 (86.00)	39 (78.00)	86 (86.00)	125 (69.44)	26 (52.00)	33 (66.00)	66 (82.50)
About the same/Somewhat/Significantly worsened	54 (30.00)	2 (4.00)	21 (42.00)	31 (38.75)	32 (16.00)	7 (14.00)	11 (22.00)	14 (14.00)	55 (30.56)	24 (48.00)	17 (34.00)	14 (17.50)
Experienced Complications that Required Urgent Attention*												
Yes	40 (22.22)	2 (2.00)	14 (28.00)	25 (31.25)	49 (24.50)	11 (22.00)	3 (6.00)	35 (35.00)	35 (19.44)	10 (20.00)	8 (16.00)	17 (21.25)
No/Not Sure	140 (77.78)	49 (98.00)	36 (72.00)	55 (68.75)	151 (75.50)	39 (78.00)	47 (94.00)	65 (65.00)	145 (80.56)	40 (80.00)	42 (84.00)	63 (78.75)

^a^The question is worded as follows in the questionnaire: How satisfied are you with the following aspects of the care experience you got most recently from this provider? (1–5 Likert scale)- traveling time

^b^The question is worded as follows in the questionnaire: How satisfied are you with the following aspects of the care experience you got most recently from this provider? (1–5 Likert scale)- access out-of-office hours by phone or text message

^c^The question is worded as follows in the questionnaire: In the past two years, did you experience at least one of these coordination problems below? A. Test results/ records not available at appointment, or duplicate tests ordered; B. Received conflicting information from different doctors; C. Specialist lacked medical history, or regular doctor not informed about specialist care

^d^This variable is the summary of the following items: quality of care (equipment), quality of care (providers), patient-centered care, out-of-pocket cost, insurance plan requirement, choices of prescription drugs, traveling time, appointment time, waiting time, opening hours, access out-of-office hours by phone or text message, coordination of needed services, comprehensiveness of services available or provided, referral from friends/relatives, and referral from a doctor (15–75 scores)

^e^This variable is worded as follows in the questionnaire: How satisfied are you with the following aspects of the care experience you got most recently from this provider? (1–5 Likert scale)-Overall satisfaction with the care experience

*p < 0.05 **p < 0.01, ***p < 0.001 based on ANOVA for continuous measures and Chi square test for categorical measures

### Analysis

The overall aim of the analysis was to compare the quality and value of care by chronically-ill patients among three cities. We performed descriptive, bivariate, and multivariate analyses. First, we used Chi-square test to compare demographic and health profiles among subjects from three cities as well as across different health care settings, and used ANOVA to compare reasons for choosing the current health providers reported by patients from the three cities. Next, we conducted bivariate analysis to compare quality and value of care indicators among subjects from the three cities, and performed ANOVA to compare the satisfaction scores of 13 indicators reported by patients from the three cities. Lastly, we applied multivariate linear regression (on continuous measures) and multivariate logistic regressions (on dichotomous measures) to test the association between models of primary care delivery and quality as well as value of chronic care after controlling for patients’ demographic and health status characteristics. We have set the significant level at 0.05 for the bivariate and multivariate analyses.

## Results

### Patient characteristics

Table 1 compares demographic and health profiles among study subjects from the three cities. Overall, a greater proportion of patients were females in Shenzhen (55.56 %), while the proportion of male was almost equal to female in Dongguan (49.00 %) and lower than female in Guangzhou (44.00 %). The average age of the participants was 62–65 and most were married. Most of the subjects in Dongguan and Guangzhou were residents but a sizable from Shenzhen were migrants. Most subjects in Shenzhen and Guangzhou were retired but a sizable from Dongguan were farmers. The education level in Shenzhen and Guangzhou was higher than in Dongguan: 46.50 % from Dongguan had primary school or below education, compared to only 25 % in either Shenzhen or Guangzhou. Per capita annual income was highest in Shenzhen, followed by Guangzhou and Dongguan (RMB 36,639.6, 26,119, 16,791.11, respectively). Most of the study subjects in Shenzhen and Guangzhou were covered under urban social insurance for workers (42.78 and 47.78 %) but a sizable from Dongguan (45.50 %) had urban social insurance for residents or other source of insurance (36.50 %). In terms of health status, patients from Guangzhou (70.56 %) were more likely to consider themselves as of fair/poor health compared to those from Shenzhen (54.44 %) and Dongguan (45.00 %). Most patients had hypertension or diabetes for their chronic conditions.

**Fig. 1:**
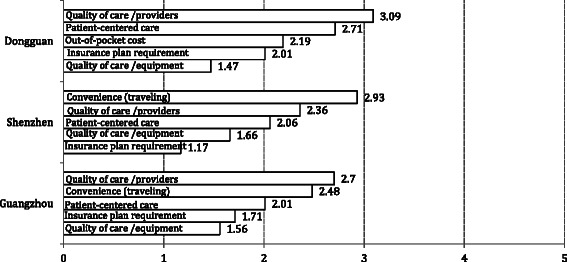
Top Five Reasons of Choosing This Facility

Figure 1 shows the top five reasons for choosing the current health providers reported by patients from the three cities. The respondents chose the top five reasons from 15 options presented in the questionnaire. The figure depicts the scores on a scale from 1 to 5 with the top reason coded as 5, the next important reason coded 4, and so on. Patients from Shenzhen and Guangzhou had comparable top five reasons despite slight difference in ranking. These were convenience (traveling), quality of care (providers), patient-centered care, quality of care (equipment), and insurance plan requirement. Patients from Shenzhen reported traveling time as their top reason for choosing this facility for care, whereas patients from Guangzhou reported quality of care (provider) as their top reason. For Dongguan, patients shared four of the five reasons as those reported by patients from Shenzhen and Guangzhou. Instead of traveling time, they identified out-of-pocket cost as one of their top five reasons.

### Quality of care

The first part of Table 2 shows 12 quality indicators that measure accessibility, continuity, coordination, and comprehensiveness of services. In general, patients from Shenzhen and Dongguan performed better compared with those from Guangzhou, and patients from Dongguan stood out on the coordination and comprehensiveness measures. Specifically, patients from Dongguan reported superior results with rate above 90 % on the following indicators: coordinate your use of medications (coordination), health care professionals talked with you about healthy diet or exercise (comprehensiveness), and not experienced coordination problems (continuity). In terms of coordination, 92 % patients from Dongguan did not experience coordination problems while the rate in Guangzhou was 81.67 %. Similarly, health care providers in Dongguan were more likely to make referral (48.00 vs. 28.33 % in Shenzhen and 13.33 % in Guangzhou), and to coordinate use of medications (95.50 vs. 80.00 % in in Shenzhen and Guangzhou). In terms of comprehensiveness, Dongguan also had significantly higher rates than Shenzhen and Guangzhou on the indicators of receiving secondary prevention services (44.00 vs. 28.89 and 31.11 %), and health professionals talking with you about things that can cause stress (47.00 vs. 39.44 and 20.56 %). Similarly, data from Table 2 also indicates significantly better performance on the access and continuity measures in Dongguan than Shenzhen and Guangzhou. In terms of the differences between types of settings, the patients in CHCs were more likely to be satisfied with traveling time in all these three cities.

**Fig. 2:**
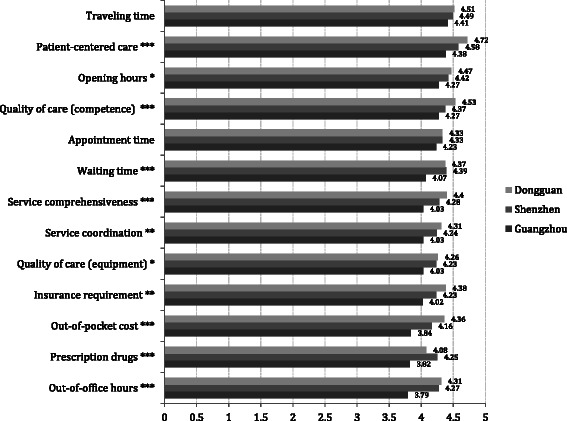
Patient Satisfaction to Current Care Provider (1–5 Likert Scale)

The relationship between models of primary care delivery and patient satisfaction with the current care provider is displayed in Fig. 2. The question is worded as follows in the questionnaire: How satisfied are you with the following aspects of the care experience you got most recently from this provider (1–5 Likert scale)? The figure visualizes the satisfaction scores of 13 indicators reported by patients from the three cities on a scale from 1 to 5 with 1 indicating least satisfied and 5 most satisfied. From the results of ANOVA analysis, patients from Dongguan reported significantly higher scores in nine of the 13 indicators (all the measures were above 4.00), greater than those from Shenzhen and Guangzhou. The most notable differences were between subjects from Dongguan and Guangzhou in service comprehensiveness (4.40 vs. 4.03, *p* < 0.001), out-of-pocket cost (4.36 vs. 3.84, *p* < 0.001), and out-of-office hours (4.31 vs. 3.79, *p* < 0.001).

We fit multivariate logistic regression models to examine patient and institutional factors associated with quality of care for the chronic disease, controlling for patient demographic and health status characteristics (Table 3). Significant associations between types of primary care delivery and all the quality indicators were observed, with the exception of satisfaction to traveling time and receiving secondary prevention. The results indicated that respondents in Dongguan were more likely to perform well on the quality indicators that measure accessibility, continuity, coordination, and comprehensiveness of services. Specifically, the probability of patients in Dongguan getting medical care in the evenings/weekends/holidays increased by 6.087 times (*p* < 0.001) compared with patients in Guangzhou, and patients in Shenzhen had 3.314 times greater odds than those in Guangzhou. Patients from Dongguan and Shenzhen were more likely to report satisfaction to access out-of-office hours by phone or text message (OR: 2.711 and 1.821 respectively; 95 % CI: 1.655, 4.441 and 1.230, 2.932 respectively). In terms of continuity, health care professionals in Dongguan were more likely to review all the medications (OR: 2.483; 95 % CI: 1.543, 3.997), to encourage patients to ask questions (OR: 3.468; 95 % CI: 2.130, 5.647), and to contact patient to follow-up with care (OR: 5.482; 95 % CI: 3.282, 9.158). Similar to the results from the bivariate analyses, patients from Dongguan reported significantly better quality of care in the domains of coordination and comprehensiveness in the multivariate analyses. The significant associations between type of health care facilities and quality of care were also observed. The results showed that patients in CHCs were more likely to be satisfied with traveling time (OR: 2. 595; 95 % CI: 1.788, 3.766).

**Table 3:** Multivariate analysis: patient and institutional factors associated with quality of care for the chronic disease

	OR (95 % CI)					
	Access			Continuity		
	Get medical care in the evenings, on weekends, or holidays	^a^Convenience (traveling time)	^a^Accessibility (access out-of-office hours by phone or text message)	Healthcare professional reviewed with you all the medications	Health professionals always/ often encourage you to ask questions	Healthcare professional contacts you to see how things are going
City						
Guangzhou	1	1	1	1	1	1
Shenzhen	3.31*** (2.06 5.34)	1.02 (0.64 1.62)	1.82* (1.13 2.93)	1.04 (0.66 1.63)	2.66*** (1.67 4.23)	3.92*** (2.43 6.32)
Dongguan	6.09*** (3.52 10.53)	1.40 (0.86 2.30)	2.71*** (1.66 4.44)	2.48*** (1.54 4.00)	3.47*** (2.13 5.65)	5.48*** (3.28 9.16)
CHC vs. hospital	1.13 (0.84 1.89)	2.60*** (1.79 3.77)	0.97 (0.67 1.41)	0.89 (0.62 1.27)	0.81 (0.56 1.16)	0.66* (0.45 0.97)
Age (Mean)	1.00 (1.00 1.02)	1.00 (1.00 1.00)	1.00 (1.00 1.00)	1.00 (1.00 1.00)	1.00 (1.00 1.00)	1.00 (1.00 1.00)
Gender						
Female vs male	1.00 (0.97 1.02)	0.99 (0.89 1.10)	1.00 (0.95 1.05)	0.99 (0.95 1.04)	0.76 (0.53 1.10)	0.74 (0.50 1.08)
Marital Status						
Married vs. not married	1.35 (0.73 2.51)	1.29 (0.72 2.29)	0.65 (0.37 1.16)	0.69 (0.39 1.22)	0.78 (0.44 1.41)	0.80 (0.44 1.48)
Residence Status						
Registered resident vs. non-registered resident	1.31 (0.83 2.06)	1.00 (0.66 1.52)	1.23 (0.81 1.86)	0.80 (0.53 1.20)	1.03 (0.68 1.55)	1.52 (0.99 2.33)
Current Occupation						
Retired vs. others	0.72 (0.45 1.13)	1.61* (1.07 2.40)	1.31 (0.87 1.96)	1.03 (0.70 1.53)	1.31 (0.87 1.96)	1.40 (0.92 2.15)
Highest Education						
Middle school or above vs. primary school or below	1.10 (0.70 1.73)	0.84 (0.56 1.27)	1.70* (1.13 2.58)	0.85 (0.58 1.26)	1.14 (0.75 1.71)	1.28 (0.83 1.97)
Per Capita Income RMB	1.00 (1.00 1.00)	1.000 (1.00 1.00)	1.00* (1.00 1.00)	1.00 (1.00 1.00)	1.00 (1.00 1.00)	1.00 (1.00 1.00)
Current Health Status						
Excellent/very good/good vs. fair/poor	2.20*** (1.44 3.38)	0.53** (0.36 0.78)	0.64* (0.44 0.93)	0.65* (0.45 0.94)	1.64* (1.13 2.38)	1.29 (0.87 1.91)
Number of Chronic Conditions	1.12 (0.90 1.39)	0.91 (0.75 1.11)	0.83 (0.68 1.02)	0.87 (0.72 1.06)	1.10 (0.91 1.35)	1.10 (0.89 1.35)
	OR (95 % CI)					
	Coordination			Comprehensiveness		
	Coordinate your use of medications	Make referrals	Didn’t experienced coordination problems	Received Secondary Prevention Services	Health professionals talked with you about things that can cause stress	Discuss with you about diet or exercise
City						
Guangzhou	1	1	1	1	1	1
Shenzhen	1.11 (0.63 1.95)	2.22** (1.25 3.95)	0.60 (0.34 1.05)	0.90 (0.55 1.46)	2.32** (1.41 3.81)	2.62** (1.29 5.31)
Dongguan	3.99** (1.76 9.05)	4.50*** (2.55 7.95)	3.52** (1.69 7.34)	1.46 (0.90 2.38)	3.65*** (2.18 6.11)	4.30** (1.86 9.93)
CHC vs. Hospital	0.55* (0.32 0.93)	0.86 (0.57 1.29)	0.85 (0.52 1.38)	1.43 (0.98 2.09)	0.99 (0.68 1.45)	0.45* (0.24 0.84)
Age (Mean)	1.01 (0.98 1.04)	1.00 (1.00 1.00)	1.00 (1.00 1.00)	1.00 (1.00 1.00)	1.00 (1.00 1.00)	1.00 (1.00 1.01)
Gender						
Female vs male	1.00 (0.98 1.03)	0.88 (0.58 1.32)	1.39 (0.85 2.28)	0.74 (0.51 1.08)	1.00 (0.95 1.04)	1.01 (0.96 1.05)
Marital Status						
Married vs. not married	0.61 (0.24 1.57)	2.19 (1.03 4.66)	0.93 (0.42 2.04)	0.84 (0.47 1.51)	1.14 (0.61 2.14)	0.74 (0.29 1.91)
Residence Status						
Registered resident vs. non-registered resident	1.83 (1.05 3.20)	1.31 (0.82 2.08)	0.93 (0.54 1.61)	1.27 (0.82 1.95)	0.84 (0.55 1.28)	1.76 (0.92 3.34)
Current Occupation						
Retired vs. others	0.46* (0.24 0.90)	0.48** (0.32 0.74)	1.16 (0.68 2.00)	0.88 (0.59 1.33)	0.84 (0.57 1.26)	1.33 (0.69 2.55)
Highest Education						
Middle school or above vs. primary school or below	1.07 (0.59 1.95)	0.74 (0.47 1.16)	1.15 (0.67 1.99)	0.91 (0.60 1.37)	1.59* (1.04 2.43)	1.41 (0.75 2.64)
Per Capita Income RMB	1.00 (1.00 1.00)	1.00 (1.00 1.00)	1.00** (1.00 1.00)	1.00 (1.00 1.00)	1.00 (1.00 1.00)	1.00 (1.00 1.00)
Current Health Status						
Excellent/very good/good vs. fair/poor	1.19 (0.70 2.04)	0.96 (0.64 1.46)	1.49 (0.88 2.50)	1.33 (0.91 1.95)	0.90 (0.62 1.32)	1.46 (0.77 2.74)
Number of Chronic Conditions	1.12 (0.85 1.48)	1.16 (0.93 1.45)	0.98 (0.76 1.26)	1.09 (0.90 1.34)	0.96 (0.78 1.17)	0.92 (0.68 1.24)

^a^We coded these two variables as binary response: score 5 in 1–5 Likert scale is coded as Yes, score 1–4 in Likert scale are coded as No

*p < 0.05 **p < 0.01, ***p < 0.001

### Value of care

Value of care was measured by satisfaction with care, concern over cost, and overall health improvement. The second part of Table 2 compares patients from the three cities on these three aspects of value. First, in terms of satisfaction, respondents from Dongguan reported significantly higher total satisfaction score and overall score (61.40 and 4.39 respectively) than those from Shenzhen (60.46 and 4.23, respectively) and Guangzhou (57.42 and 4.16, respectively). Second, in terms of cost, compared with patients from Shenzhen and Guangzhou, patients from Dongguan were more likely to be satisfied with the out-of-pocket cost for their chronic care (49.40 vs. 30.19 and 27.13 %, *p* < 0.001). Third, in terms of health improvement, compared with patients from Shenzhen and Guangzhou, patients from Dongguan were more likely to report improvement with their chronic condition relative to when it was first diagnosed (84.00 vs. 70.00 and 69.44 %, *p* < 0.01).

Table 4 shows the results of multivariate analyses of patient and institutional factors associated with value of care for the chronic disease, controlling for patient demographic and health status characteristics. We fit multivariate linear regression models to examine patient and institutional factors associated with total and overall scores of satisfaction with care. Similar to the results from the bivariate analyses, patients from Dongguan reported significantly higher total score (*p* < 0.001) as well as overall score of satisfaction with care (*p* < 0.05), compared to those from Guangzhou. In particular, patients from Dongguan scored an average of 4.354 more points on total satisfaction score and an average of 0.206 points higher on the overall satisfaction score than those from Guangzhou. The rest part of Table 4 displays the multivariable logistic regression results examining factors associated with cost concern and health improvement with the chronic condition. Significant associations were observed between models of primary care delivery and concern over cost as well as overall health improvement. Specifically, the probability of patients from Dongguan satisfied with out-of-pocket cost for chronic care increased by 2.889 times (*p* < 0.01) compared with patients from Guangzhou. Patients from Dongguan were also more likely to indicate improvement in their chronic condition relative to when it was first diagnosed (OR: 2.221; 95 % CI: 1.257, 3.925).

**Table 4:** Multivariate analysis: patient and institutional factors associated with value of care for the chronic disease

	Estimates (SE)		OR (95 % CI)			
	Satisfaction		Cost Concern		Health Improvement	
	Total Score of Satisfaction of Current Care Provider	Overall Satisfaction with the Care Experience	Satisfaction to Out-of-pocket Cost for Chronic Care	Receive the Help You Needed Despite the Cost	Chronic Condition Relative to When it was First Diagnosed	Experienced Complications that Required Urgent Attention
City						
Guangzhou	1	1	1	1	1	1
Shenzhen	3.55 (0.80)***	0.07 (0.08)	0. 95 (0.49 1.82)	0.89 (0.41 1.93)	0.79 (0.48 1.28)	1.48 (0.83 2.64)
Dongguan	4.35 (0.84)***	0.21 (0.09)*	2.90** (1.38 6.03)	0.85 (0.40 1.82)	2.22** (1.26 3.93)	2.00* (1.10 3.68)
CHC vs. Hospital	−0.72 (0.64)	−0.18 (0.07)**	1.40 (0.82 2.41)	0.76(0.42 1.37)	0.93 (0.62 1.41)	0.44*** (0.28 0.69)
Age (Mean)	−0.01 (0.003)	−0.001 (0.0004)**	1.01 (0.98 1.04)	1.00 (1.00 1.00)	1.00 (1.00 1.01)	1.00 (1.00 1.00)
Gender						
Female vs male	−0.01 (0.01)	−0.0002 (0.0007)	1.01 (0.97 1.04)	1.00 (1.00 1.03)	1.00 (1.00 1.04)	0.76 (0.48 1.19)
Marital Status						
Married vs. not married	−0.30 (1.01)	−0.06 (0.10)	0.43 (0.15 1.22)	0.36 (0.11 1.22)	0.63 (0.32 1.27)	0.78 (0.40 1.52)
Residence Status						
Registered resident vs. non-registered resident	1.45 (0.72)*	0.16 (0.07)*	1.63 (0.90 2.96)	0.98 (0.50 1.93)	0.56 (0.34 0.92)	1.38 (0.87 2.30)
Current Occupation						
Retired vs. others	1.52 (0.70)*	−0.04 (0.07)	1.21 (0.65 2.27)	1.58 (0.86 2.88)	0.72 (0.45 1.15)	1.07 (0.65 1.77)
Highest Education						
Middle school or above vs. primary school or below	0.62 (0.70)	−0.03 (0.07)	1.77 (0.95 3.32)	1.36 (0.75 2.48)	1.64* (1.05 2.56)	1.10 (0.66 1.84)
Per Capita Income RMB	−0.00002 (0.00001)	0.000001 (0.000001)	1.00 (1.00 1.00)	1.00* (1.00 1.00)	1.00 (1.00 1.00)	1.00 (1.00 1.00)
Current Health Status						
Excellent/very good/good vs. fair/poor	0.05 (0.66)	0.06 (0.07)	1.55 (0.87 2.74)	0.79 (0.44 1.42)	1.43 (0.92 2.22)	0.67 (0.42 1.07)
Number of Chronic Conditions	−0.70 (0.34)*	−0.01 (0.04)	0.60*** (0.45 0.79)	0.64** (0.48 0.84)	0.90 (0.73 1.11)	1.93*** (1.53 2.42)

*p < 0.05 **p < 0.01,***p < 0.001

## Discussion

This study was one of the first to examine the impact of models of CHC-hospital arrangements on access to and quality of care for patients with chronic illness in China. The study provided evidence that appropriately designed primary care delivery could enhance access, improve quality, and provide value to patients with chronic illness. First, the results from this study showed that patients from all three cities chose their current health care providers primarily out of concern for quality of care (both providers and equipment), patient-centered care, and insurance plan requirement. Therefore, enhancing quality at CHCs both in terms of provider skills and medical equipment is critical to attracting and retaining patients. The provision of insurance plan also facilitates the use of CHCs.

Next, compared with patients from Guangzhou, those from Dongguan performed significantly better on most quality and value of care indicators. Most of these indicators (16 out of 18) were still significantly better (at the magnitude of 1.5 to 6.1 times) even after controlling for patients' demographic and health status characteristics. Particularly, the results showed large effect size in the indicators of getting medical care in the evenings/weekends/holidays, health care professional contacting patients to follow-up with care, making referral, and total score of satisfaction with the current care provider. These impressive results suggested that the Dongguan model (based on insurance mandate and using family practice physicians as ‘gate-keepers)’ seemed to work best in terms of improving access and quality for patients with chronic conditions. The higher performance in accessibility suggested that the compulsory gate-keeping arrangement that stipulated patients start their treatment at CHCs close to their living place worked in guiding patients to appropriate medical institutions based on the severity of diseases. The higher performance in the coordination domain suggested that the Dongguan model successfully integrated health service at different levels of health care system under the government’s ownership and management [56, 58]. The higher performance in the continuity of care and comprehensiveness of services domains confirmed the efficacy of the family practice physicians that Dongguan CHCs relied on in serving their patients. As consistent with previous studies, primary care system with gatekeepers was associated with better quality of care and affordable medical cost [59, 60].

Although results from Shenzhen were less impressive than Dongguan, they were still significantly better than Guangzhou, recording 8 significant indicators out of 18 even after controlling for patients' demographic and health characteristics. These results suggested that the Shenzhen model (hospital-owned and -managed CHC) was generally effective in enhancing accessibility and continuity. Coordination was less impressive presumably due to duplicating primary care services at the hospital setting in Shenzhen. Since primary care outpatients at the hospital contributed a significant proportion of the hospital revenue, the hospital and CHC were in a somewhat competitive (rather than collaborative) position for patients thus hampering referrals.

Of the three models, the Guangzhou model (allowing patients to choose providers and settings) seemed to fare the worst. Given a choice, most Chinese still prefer large hospitals out of habit as well as the perception of better quality. To channel patients to CHCs for their primary care, insurance mandate seems essential, along with the improvement in practice quality (as seen in the Dongguang model). Although the Dongguang model suggested that having a USC can improve quality of primary care, this is not yet a requirement in China and the government imposes no restrictions on health care provider selection. Because of this, health resources may not be effectively used, as patients will crowd in tertiary hospital although their illnesses are not so serious. This may not only reduce the quality of primary care patients receive, but also a waste of health resources. Our study suggested that if there were a health policy guiding patients to use a usual source of care (USC), the overall quality of primary care might improve and the use of health resources could be more appropriate. In addition to promoting the gatekeeper role of primary care doctors, the other potential methods to improve patients seeking primary care at appropriate levels include expanding primary care infrastructure, offering financial/insurance incentives, establishing two-way referral system, and collaborating with the community to initiate outreaching health programs. For example, in order to make care accessible financially and geographically, Spain has enacted universal insurance coverage and expanded primary care infrastructure to meet the goals that there be a primary care center within a fifteen-minute radius of any place of residence [61]. Moreover, to address the barriers associated with controlling chronic conditions such as inadequate follow-up of treatment, lack of support for self-management, patient’s failure to adhere to treatment, culturally based differences in perceptions of health, and costs of transportation and other expenses, CHCs can be called upon to play a crucial role in providing culturally appropriate, timely and accessible care, supporting patient self-management, providing outreach to patients in the community, educating patients on the importance of lifestyle changes and adherence to their medication, and promoting continuity of care.

The current study had several limitations. First, the cross-sectional nature of the study made it difficult to make causal inferences from the analyses. Second, the study sites were selected only from one province, which limited the representativeness and generalizability of the study results. And the heterogeneity of the three field sites could have influenced the results of the study. Further research is needed to expand the investigation among multi-sites and to conduct prospective and experimental studies, such as using randomized clinical trials design. Third, the study examined patients’ self-perceived experiences rather than clinical or other more objective health outcomes. Future analyses could include clinical data to examine the health outcomes among patients with specific chronic illness.

Despite these limitations, findings from this study are helpful in informing policy decisions and practice. This study is among the first to examine the association between new primary care models and quality as well as value of care in China, providing an understanding of the impact of these new models on access and coordination of care for older patients with chronic conditions, and making suggestions for improving chronic care at appropriate levels of the system. To face the challenges of a rapidly aging population and eruption of non-communicable disease epidemic, an adequately funded and well-organized primary care system can play a gatekeeping role and has the potential to provide a reasonable level of care to patients.

## Abbreviations

CHCs: Community health centers
USC: Usual source of care

## Footnotes

1 Source: Data form Guangzhou, Shenzhen and Dongguan.
